# Semi-automated myocardial segmentation of bright blood multi-gradient echo images improves reproducibility of myocardial contours and T2* determination

**DOI:** 10.1007/s10334-016-0601-0

**Published:** 2016-12-16

**Authors:** Pandji Triadyaksa, Niek H. J. Prakken, Jelle Overbosch, Robin B. Peters, J. Martijn van Swieten, Matthijs Oudkerk, Paul E. Sijens

**Affiliations:** 1 Center for Medical Imaging-North East Netherlands, University of Groningen, University Medical Center Groningen, EB45, 30001, 9700 RB Groningen, The Netherlands; 2 Department of Radiology, University of Groningen, University Medical Center Groningen, EB45, 30001, 9700 RB Groningen, The Netherlands; 3 Department of Physics, Diponegoro University, Sudharto Street, Semarang, 50275 Indonesia

**Keywords:** Magnetic resonance imaging, Iron loading, Bright blood myocardial T2*, k-Means clustering, Vector field convolution active contour

## Abstract

**Objectives:**

Early detection of iron loading is affected by the reproducibility of myocardial contour assessment. A novel semi-automatic myocardial segmentation method is presented on contrast-optimized composite images and compared to the results of manual drawing.

**Materials and methods:**

Fifty-one short-axis slices at basal, mid-ventricular and apical locations from 17 patients were acquired by bright blood multi-gradient echo MRI. Four observers produced semi-automatic and manual myocardial contours on contrast-optimized composite images. The semi-automatic segmentation method relies on vector field convolution active contours to generate the endocardial contour. After creating radial pixel clusters on the myocardial wall, a combination of pixel-wise coefficient of variance (CoV) assessment and *k*-means clustering establishes the epicardial contour for each segment.

**Results:**

Compared to manual drawing, semi-automatic myocardial segmentation lowers the variability of T2* quantification within and between observers (CoV of 12.05 vs. 13.86% and 14.43 vs. 16.01%) by improving contour reproducibility (*P* < 0.001). In the presence of iron loading, semi-automatic segmentation also lowers the T2* variability within and between observers (CoV of 13.14 vs. 15.19% and 15.91 vs. 17.28%).

**Conclusion:**

Application of semi-automatic myocardial segmentation on contrast-optimized composite images improves the reproducibility of T2* quantification.

## Introduction

Cardiovascular magnetic resonance imaging (MRI) techniques are used for non-invasive assessment of patients with iron loading in the heart, i.e. thalassemia, hemochromatosis, cardiomyopathy and sickle cell disease [1–5]. The assessment includes multi-gradient echo (MGE) imaging to quantify myocardial T2* in bright blood as well as black blood modes [3–7]. Recent assessments of iron loading feature the mid-ventricular septum as well as other areas of the myocardium [1, 8, 9].

Myocardial iron loading identification at an early stage is important to prevent cardiac complication [8] leading to heart failure [10]. Assessment of the entire myocardium rather than just the septum enables the prediction of iron loading at an early stage [1, 8], also considered relevant for the characterization of pathology in post-mortem studies [11–14]. In the global assessment, susceptibility artifacts are frequent in posterior lateral and anterior regions, but may be corrected for to ensure reliable myocardial T2* values [15–18].

In the assessment of myocardial iron deposition, manual myocardial contour drawing is time-consuming and subject to intraobserver and interobserver variability. Several segmentation methods have been developed to produce automatic left ventricular (LV) myocardial contours [19], but their accuracy is influenced by tissue contrast quality that depends on intrinsic tissue parameters, scanning hardware, sequence type, and imaging parameters [20]. Image-based (i.e. *k*-means clustering) and contour-based (i.e. active contours) approaches are widely used in LV myocardial segmentation but their application is sensitive to image intensity and contrast variability or requires a large training dataset to account for anatomical and imaging variations [20]. Using prior knowledge of LV morphology, the application of these two approaches has been effective to automatically determine the LV myocardial contours for various MR parameters and settings [19].

**Fig. 1:**
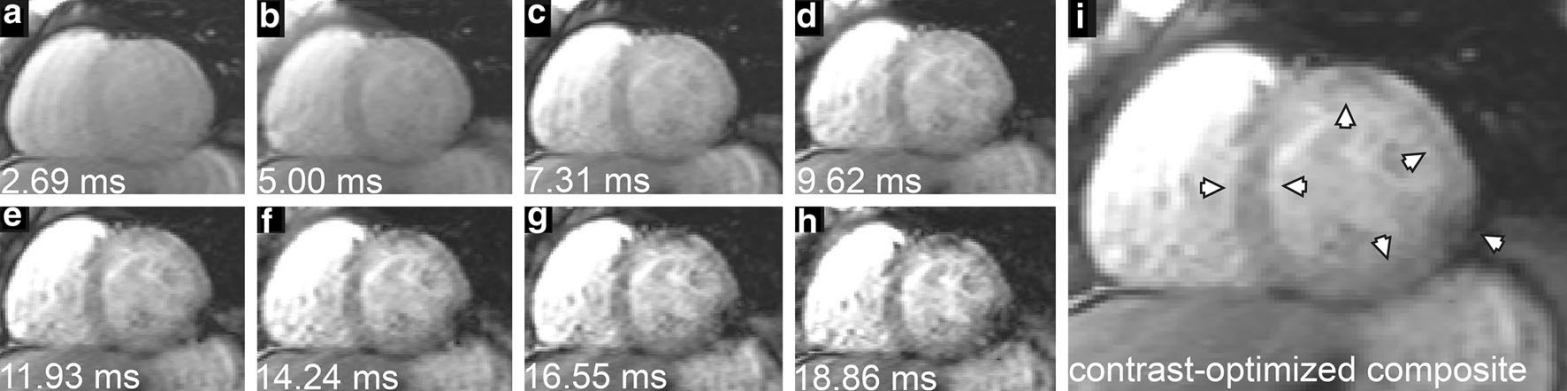
Eight short-axis images of a bright blood multi-gradient echo series (**a**–**h**) and the contrast-optimized composite image (**i**) generated by combining the image at an echo time (TE) of 2.69 ms, as a representation of optimum contrast between the myocardium and lung, 14.24 ms for optimum contrast between the myocardium and right ventricle blood pool and 18.86 ms for optimum contrast between the myocardium and left ventricle blood pool. Compared to any single TE image, contrast improvements at epicardial and endocardial borders, especially at the free wall myocardium, show on the composite image where at the inferior epicardial border gradient echo susceptibility artifacts are not prominent (contrast improvements highlighted by *arrow heads*)

Commercial software packages do feature automatic methods in their delineation option but, to our knowledge, are focused on myocardial MR cine imaging [21–25] and are not specifically designed for myocardial T2* assessment. Recently, an automatic method was presented for segmenting the mid-ventricular region for myocardial T2* measurement using black blood [17, 26] and bright blood [27] MGE series. Even though the black blood mode is more favorable for iron loading quantification [7], the bright blood mode is still widely used as a standard sequence for large populations [1, 8] and follow-up studies [28, 29]. In bright blood mode, myocardial segmentation remains a challenge due to the poor contrast between the myocardium and its surroundings on the original MGE images [19]. The use of a single image with the shortest echo time (TE) as a template for morphological operation still produces high observer variability as reported elsewhere [27]. The combination of eight MGE images acquired with different TEs to increase the detectability of the myocardial boundary information was proposed, but without documentation of the obtained contrast improvement [27]. As an alternative, a composite image offering better contrast [30] can serve as a template image. The use of a composite image (Fig. 1i) as a combination of three images representing up to three TEs from the MGE series (Fig. 1a–h) has recently been shown to reduce observer variability in drawing LV epicardial and endocardial contours [30] compared with the common practice of using a single short-axis image corresponding to a single TE [3, 7, 8, 27]. This procedure, while improving the reproducibility of myocardial contours, is still observer-dependent. This study aims to resolve the difficulties of myocardial T2* assessment in a bright blood MGE series by improving the generation of contrast-optimized composite images and use it as a template for image-based and contour-based semi-automatic segmentation, combined with prior knowledge of LV morphology, to advance the reproducibility of LV myocardial contour and T2* quantification.

## Materials and methods

### Patients

This retrospective study involved post-processing of clinical data acquired between February 2009 and September 2015, with approval from the hospital review board that waived informed consent. In this period, 22 patients were examined by a clinical routine cardiac MRI protocol including MGE with three short-axis slices at apical, mid-ventricular, and basal locations. Five patients were excluded due to motion blurring. The remaining 17 patients consisted of 6 cases of thalassemia, 6 with hemochromatosis, 3 with suspected cardiomyopathy, 1 with sickle cell disease, and 1 congenital dyserythropoietic anaemia patient.

### Multi-gradient echo magnetic resonance imaging

Cardiac MRI studies were performed at 1.5 Tesla (T) using one of two Siemens scanners (Siemens Medical Solution, Erlangen, Germany), between 2009 until 2011 the Avanto in seven patients, and from 2012 until 2015 the Aera in ten patients. A body matrix coil of 6 to 9 elements and a spine matrix coil of 12 to 24 elements were used depending on the chosen field of view (FOV). A single breath-hold bright blood MGE sequence was performed at eight TEs (2.59–18.20 at 2.23-ms increments using the Siemens Avanto and 2.69–18.86 at 2.31-ms increments using the Siemens Aera) with a repetition time (TR) of 200 ms and a flip angle of 20°. On both scanners, a variable FOV of 275–362 × 400 mm^2^ was applied according to patient size with a reconstructed voxel size of 1.56 × 1.56 × 10 mm^3^, 50% phase resolution sampling, using 18–24 cardiac cycles per breath-hold with 5 segments in each heart beat and without enabling parallel imaging. Pixel bandwidth was set at 814 Hz for the Avanto scanner and 815 Hz for the Aera scanner. From the included 17 patients a total of 51 LV short-axis slices were acquired.

### Composite image generation

**Fig. 2:**
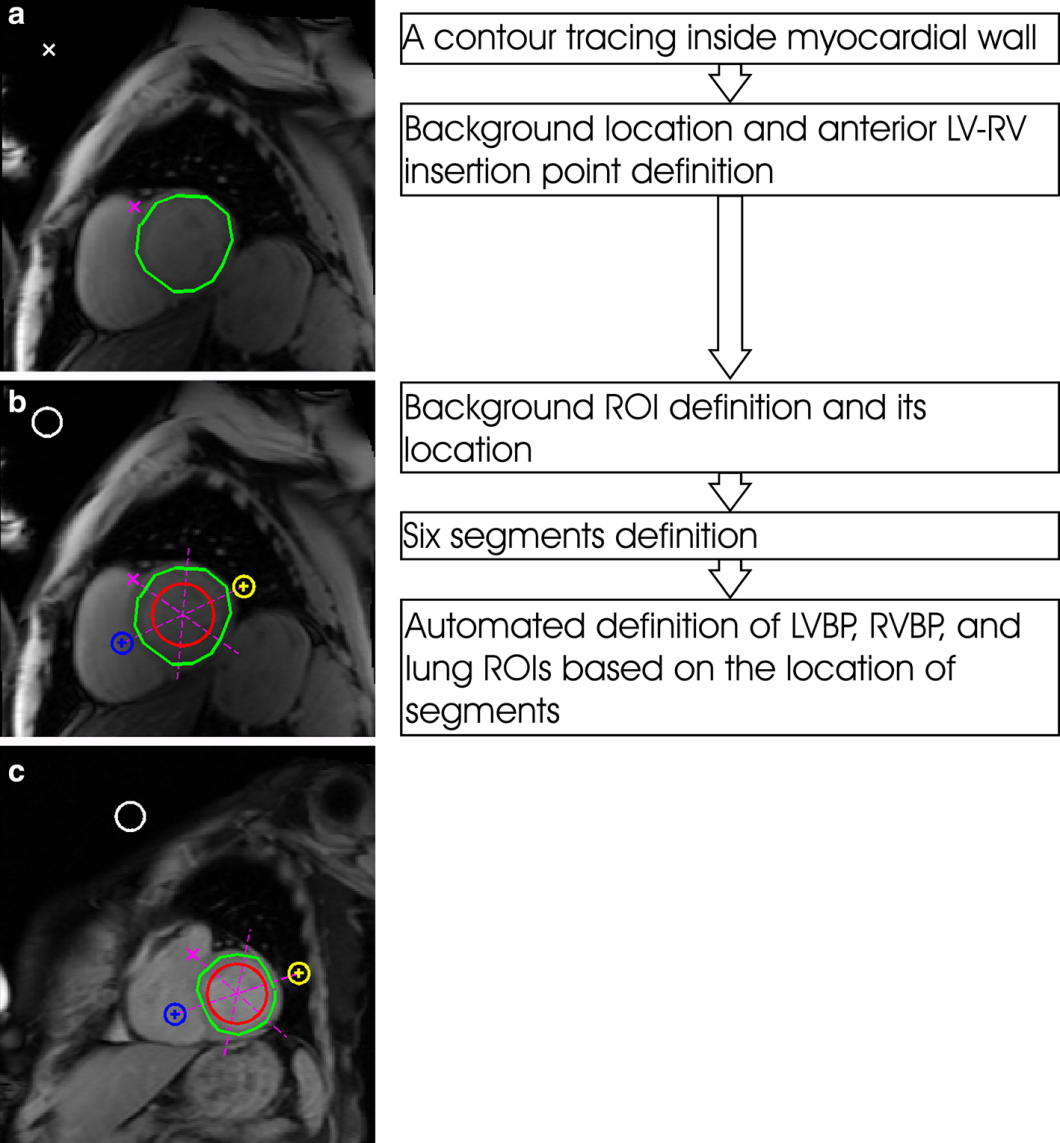
Steps of contrast-to-noise ratio calculations on multi-gradient echo images by using method 1 (**a**, **b**) to define regions of interests (ROIs) of left ventricle blood pool (LVBP), right ventricle blood pool (RVBP), and lung. An example of the ROIs generated by method 1 (**c**) is presented

**Fig. 3:**
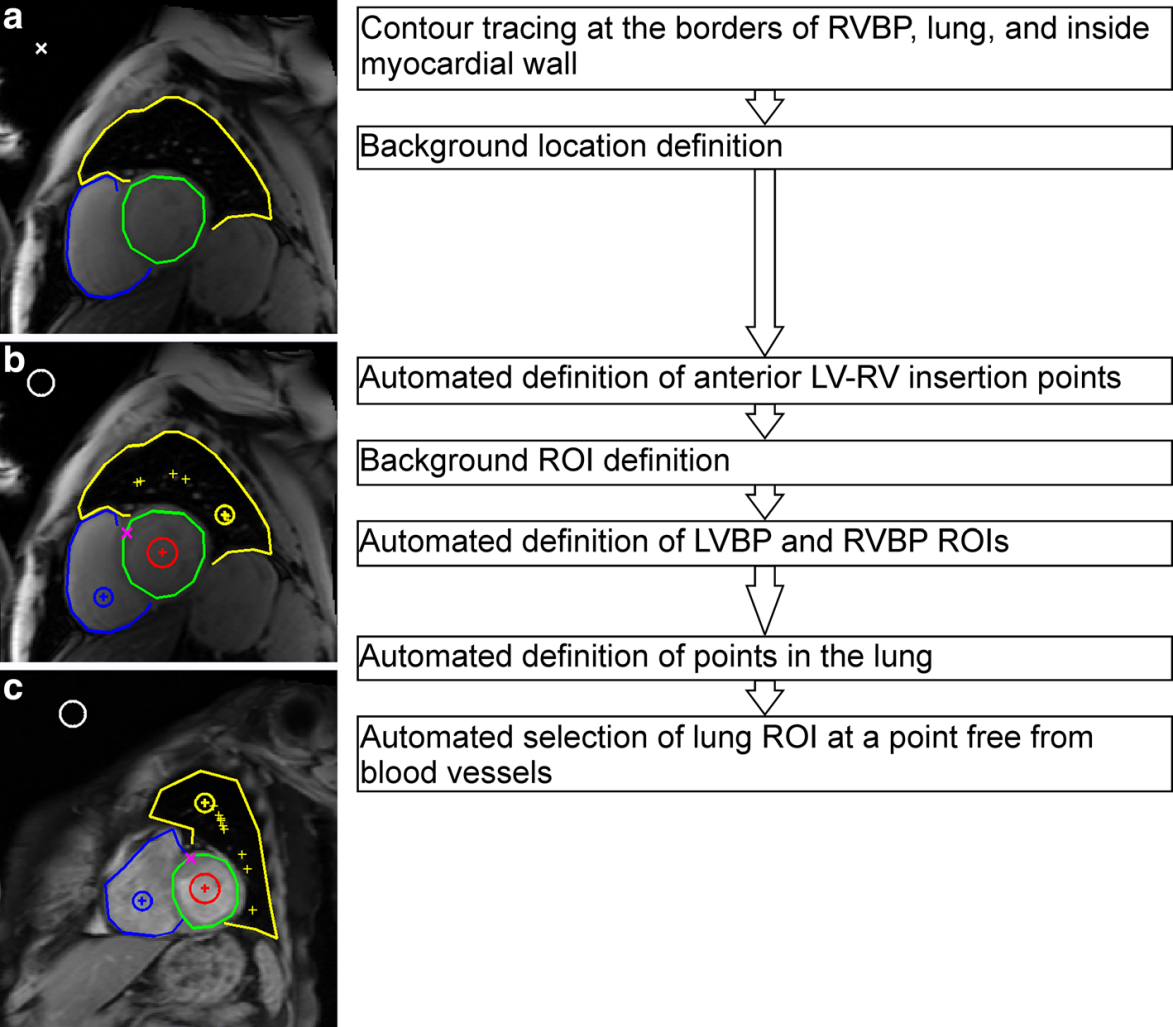
Steps of contrast-to-noise ratio calculations on multi-gradient echo images by using method 2 (**a**, **b**) to define regions of interests (ROIs) of left ventricle blood pool (LVBP), right ventricle blood pool (RVBP), and lung. An example of the ROIs generated by method 2 (**c**) is presented

In this study, the improvement of contrast-optimized composite images generation was conducted semi-automatically by two methods (Figs. 2, 3) using custom-written software (developed in MATLAB version 7.14, The MathWorks, Natick, MA, USA).

The first method involved tracing a manual contour inside the LV myocardial wall, rather than exactly on the endocardial and epicardial borders, on the shortest TE image from the MGE series to acquire the LV signal intensity along its path, locating a coordinate of air background staying clear of any visible artefact, and locating the coordinate of the anterior LV and right ventricle (RV) insertion point [18]. In Fig. 2a, this is illustrated for a mid-ventricular short axis slice. On the indicated location of air background, an ROI was automatically generated. Based on the insertion location, the LV myocardium was automatically divided into four segments (anterior, septal, inferior, and lateral) at an apical slice, and six segments (anterior, anteroseptal, inferoseptal, inferior, inferolateral, and anterolateral) at basal and mid-ventricular slices. An ROI of LV blood pool (LVBP) was automatically located at the cross-section of the segments inside the blood pool. The right ventricle blood pool (RVBP) ROI was automatically positioned by extrapolating the anteroseptal and inferoseptal segment junction into the nearby RVBP, and the lung ROI was ascertained by extrapolating the inferolateral and anterolateral segment junction into nearby lung (Fig. 2b). An exception is on the apical slice; due to the small area of the RVBP and lung, the ROIs of the RVBP and lung were generated automatically after manually marking their approximate locations. All ROIs and LV myocardial wall contours were automatically propagated through the MGE image series.

The second method required a location of air background while staying clear of imaging artefacts, manual contouring inside the LV myocardial wall, contouring the RVBP, and lung regions on the shortest TE image from the MGE series (Fig. 3a). The anterior LV and RV insertion points were established automatically at the end of the anterior RVBP contour. An ROI at the center of the LVBP was defined by the coordinate of LV myocardial contour. The complete RVBP area was identified combining the septal myocardium and the RVBP contours. The whole lung area was derived from the lateral myocardium and lung contours. The coordinates of RVBP and lung contours defined an ROI at the center of the RVBP and an ROI in the lung. At the lung, the ROI was taken in an area without blood vessels from one of several possible locations (Fig. 3b). The appointed ROIs of LVBP, RVBP, lung, and the LV myocardial wall contour were then automatically propagated through the MGE image series. This method was applied on all three (apical, mid-ventricular and basal) short-axis slices.

After acquiring the signal intensity of the LV myocardial wall, LVBP, RVBP, and lung through the MGE series, contrast-to-noise ratio (CNRs) between the LV myocardium and its surroundings were defined as follows [30]:

1 $${\text{CNR = NF}}\frac{{ | {\text{SI}}_{\text{s}} -{\text{SI}}_{\text{m}} |}}{{\sigma_{\text{b}} }}$$

where NF, SI_s_, SI_m_, and *σ*_b_ represent the noise factor [30], the signal intensities of the surroundings of the LV myocardium (LVBP, RVBP, and lung), the signal intensity of LV myocardium, and the standard deviation of air background, respectively. Next, a contrast-optimized composite image was generated from the images of optimum CNR [30]. In the example of Fig. 1, the composite image (Fig. 1i) was thus generated by combining the images of TE 18.86 ms (Fig. 1h), 14.24 ms (Fig. 1f), and 2.69 ms (Fig. 1a) that represents an optimum myocardial CNR relative to its surroundings (LVBP, RVBP, and lung).

### Semi-automatic segmentation

**Fig. 4:**
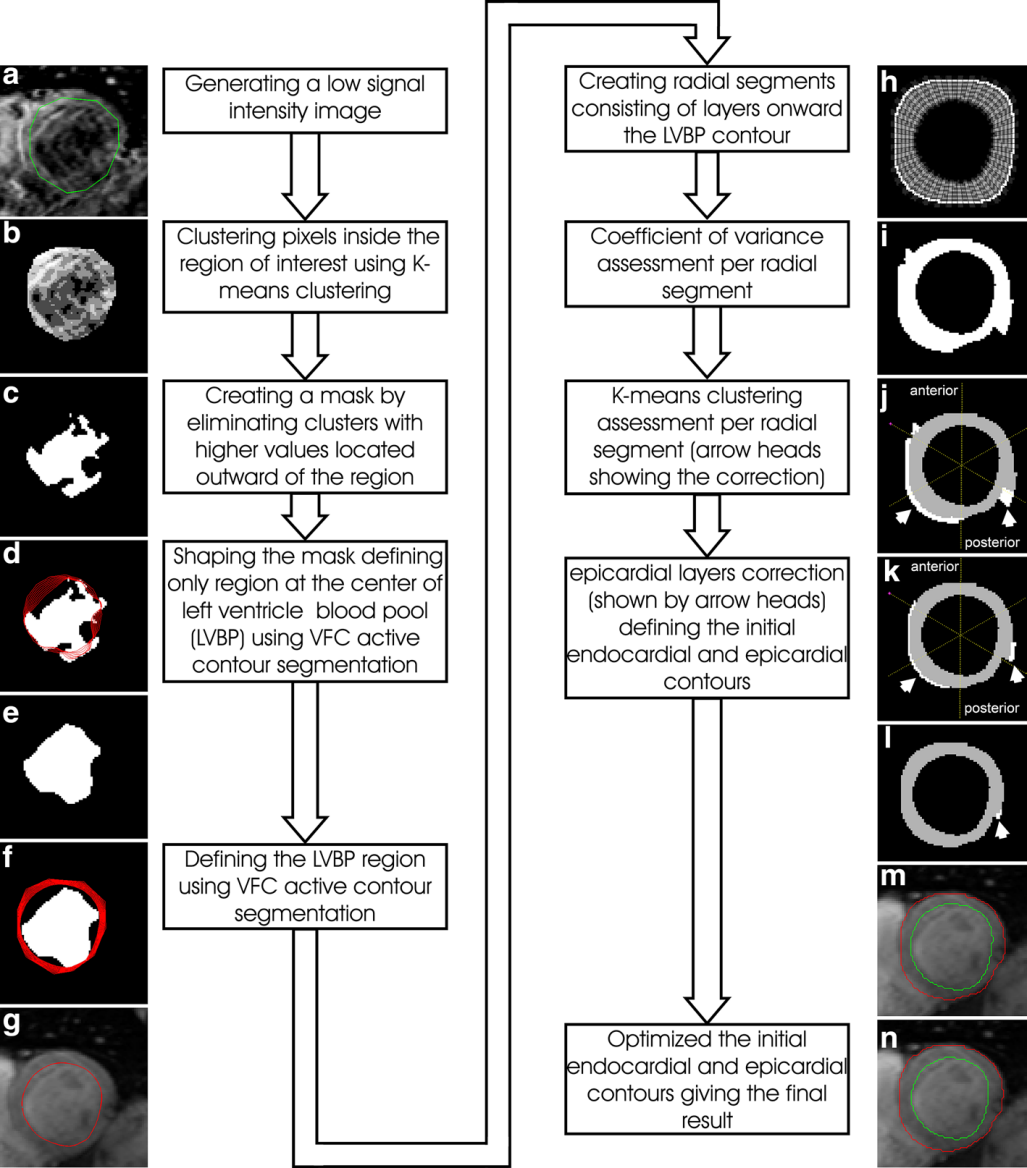
Semi-automatic myocardial segmentation process

The generation of the contrast-optimized composite images is a semi-automatic procedure, while the actual segmentation process is fully automated. The processes involve ten steps adding up into two main parts (Fig. 4).

#### Part I. LVBP region definition

This part starts by defining the LVBP location on the short-axis image. In this study featuring a bright blood MGE series, blood signal artefacts, partial volume effects, and the low contrast difference with myocardium make the center definition of LVBP difficult to determine. Therefore, a low-signal-intensity image is proposed (step 1), inspired by the use of blood signal suppression on the black blood image [7, 26], to produce signal homogeneity at the LVBP area with adequate contrast between the blood pool and myocardium. Benefiting from the different transverse magnetization decay rate of the LVBP and the myocardium, the image is created by subtracting the shortest TE image with the optimum TE between the myocardium and the LVBP (Fig. 4a). Using the ROI of the myocardium defined earlier (Figs. 2a, 3a), the center of the LVBP is then located by *k*-means clustering (step 2) [31, 32]. Using this clustering, the pixels inside the ROI are grouped based on signal intensity (Fig. 4b) and a mask of the remaining blood pool area at the center is then determined (step 3; Fig. 4c) by removing high cluster values representing myocardial tissue and any artefacts. Afterward, a vector field convolution (VFC) active contour segmentation [33, 34] is applied (step 4; Fig. 4d) to shape the blood pool region (Fig. 4e) which is used as a mask for the subsequent active contour segmentation (step 5; Fig. 4f) to determine the LVBP ROI including papillary muscles and trabeculae (Fig. 4g).

#### Part II. Myocardial borders definition

From the LVBP ROI, a mask of one-pixel-thick layers extending beyond any possible myocardial area is created by using the signal intensity of the composite image (step 6; Fig. 4h). A maximum normal short-axis LV myocardial thickness of 9.6 ± 1.6 mm at basal, 8.5 ± 1.2 mm at mid-ventricular, and 6.6 ± 1.2 mm at apical level as reported by Kawel et al. [35] is used in this study as expansion limits to create mask layers of 14 mm at basal and mid-ventricular slices, and 8 mm at maximum for apical slices. On the first layer adjacent to the LVBP ROI, 72 radial segments on the mid-ventricular and basal slices, and 40 segments on the apical slices are created to ensure that each segment is only filled by up to 2 stacks of pixels measured at the inner layer (Fig. 4h). These amounts of radial segments were chosen to detect the myocardial contour up to pixel level.

Prior knowledge of LV morphology detects signal heterogeneity at outer layers representing the area outside the myocardium. Therefore, coefficients of variance (CoV) in pixel signal intensity, expressed as a standard deviation divided by their mean, are assessed per layer of expansion at each radial segment (step 7). Two adjacent segments at the innermost layer with the smallest CoV are defined as a starting point for the CoV assessment. Initial epicardial border expansion stops as soon as the CoV of the layer’s signal intensity exceeds 20%, an empirically optimized threshold. Owing to the high difference of signal intensity between the myocardium and lung, the initial lateral epicardial border was thus located properly as shown in Fig. 4i. The initiation of the epicardial border at anterior, septal and posterior positions, inaccurate due to the low contrast difference at the border, is refined by assessing a *k*-means clustering from the layer expansion of the CoV assessment (step 8) to create two clusters. After that, the inner cluster is selected as the initial epicardial contour (radial clusters in white colour are excluded from the myocardial region as shown by the arrow head in Fig. 4j).

Further correction is applied (step 9) to eliminate false areas at outer layer(s) of anterior, septal and posterior positions that contain less than 70% of the maximum number of pixels per layer (a pixel layer in white colour is excluded as shown by left arrow head in Fig. 4k). Meanwhile, the lateral position is corrected similarly with a cut-off value of 40% (excluding two pixel layers at the right arrow in Fig. 4k). By maximizing the difference in the number of layers per radial segment relative to each neighbour at two (arrow head in Fig. 4l), initial endocardial and epicardial contours are established (Fig. 4m).

**Fig. 5:**
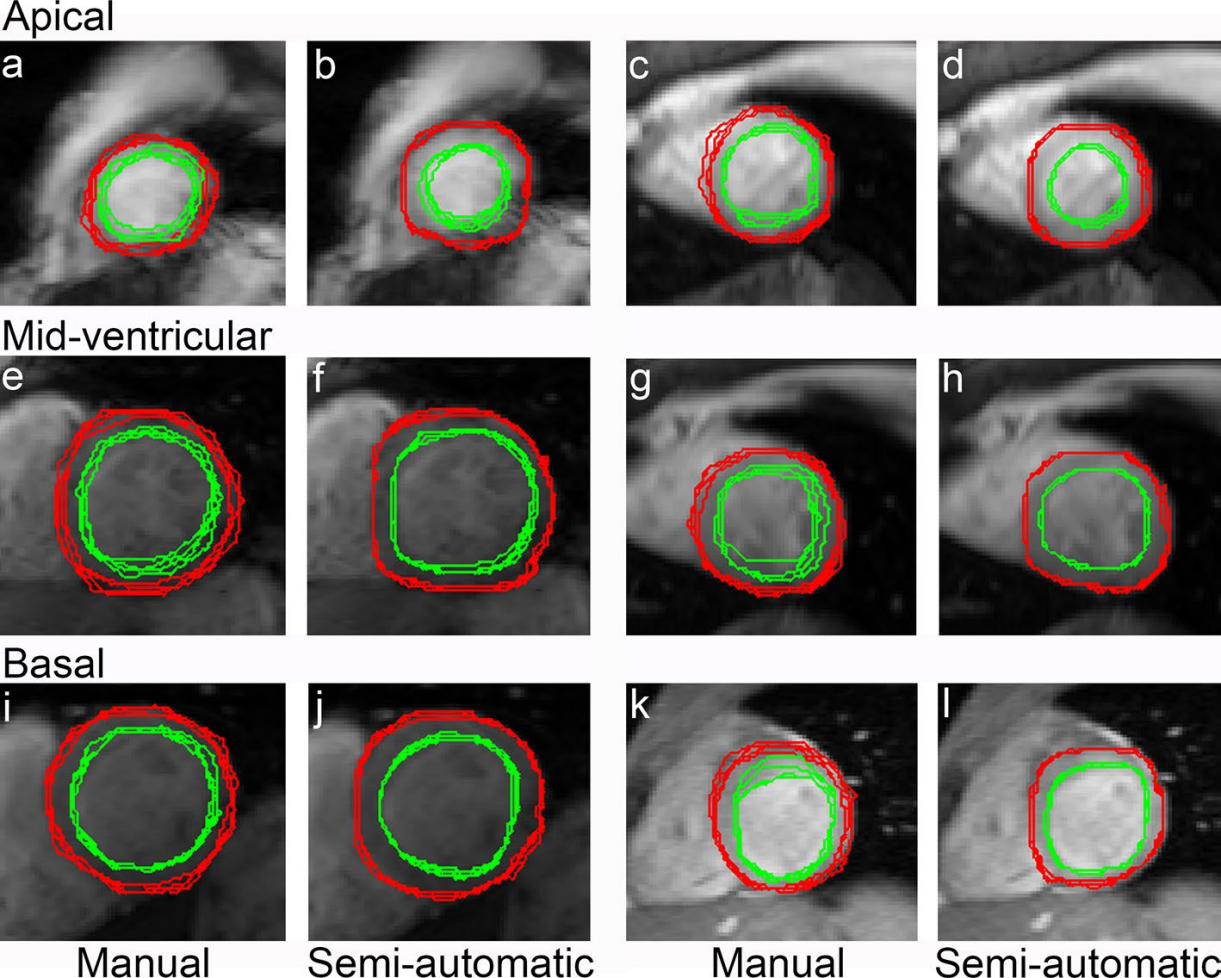
An overlay of four myocardial contours assessed by four observers on a contrast-optimized composite image generated by method 2 by manual drawing at apical (**a**, **c**), mid-ventricular (**e**, **g**) and basal locations (**i**, **k**) with its comparison by using semi-automatic segmentation (**b** and **d** at apical, **f** and **h** at mid-ventricular, and **j** and **l** at basal locations). Representative contours on slices in the presence (T2* ≤ 20 ms) and absence of iron deposition (T2* > 20 ms) in any segment can be seen in **a**, **b**, **e**, **f**, **i**, **j**, and **c**, **d**, **g**, **h**, **k**, **l**, respectively

For a more precise setting of the myocardial contours, two pixel layers inward and outward of the initial epicardial and endocardial contours are added (step 10), creating several combinations of myocardial thickness expansion and compression. The layer expansion or compression serves to test the homogeneity of myocardial area at the borders relative to the whole myocardium. For each combination, pixels in the layers having signal intensities beyond 0.9825 × Q1 of the interquartile range (IQR) and 0.9825 × Q3 of the IQR (empirically established thresholds) are counted and identified as outliers. The combination of myocardial layers having the minimum ratio of outlier pixels to total myocardial pixels is then selected as the new myocardial area at the borders. The outlier pixels in the selected layer’s combination, indicating the area outside or on the edge of the myocardium, are excluded and optimal endocardial and epicardial contours identified (Fig. 4n). In the example of Fig. 4n, optimal contours are acquired by adding one layer at the endocardial border inward to the LVBP and removing outlier pixels at the epicardial border. The optimal myocardial contours as generated by the semi-automatic segmentation are comparable with manual drawing as can be seen in Fig. 5.

### T2* quantification

A monoexponential fitting model with a constant offset was used by the software to quantify pixel-wise myocardial T2* [4]:

2 $$y = Ke^{{{\raise0.7ex\hbox{${ - {\text{TE}}}$} \!\mathord{\left/ {\vphantom {{ - {\text{TE}}} {{\text{T2}}^{ *} }}}\right.\kern-0pt} \!\lower0.7ex\hbox{${{\text{T2}}^{ *} }$}}}} + C$$

where *y*, *K*, TE, T2*, and *C* represent signal intensity, a fitting constant, echo time, myocardium transverse relaxation time, and a constant of offset correction, respectively. Monoexponential fitting with a constant is known to reduce underestimation of T2* values at higher iron concentrations, both in pixel-wise, and region-based quantifications [4]. Pixel-wise T2* was calculated for four apical, six mid-ventricular and six basal segments, according to the American Heart Association (AHA) 16-segment model [8] using median rather than mean T2* values to reduce the influence of pixel-wise noise and artefact errors [36, 37]. The AHA segments were defined by using the anterior LV and RV reference points of the composite image generation. Global T2* was defined as an average value over 16 segments, and the mid-ventricular septal T2* was the average T2* value of the mid-anterior septum and mid-inferior septum segments.

### Reproducibility assessment of myocardial segmentation

By using the custom-written software as described above, two radiologists (NHJP and JO), one radiology resident (RBP), and one experienced MR technician (JMvS), generated the contrast-optimized composite images by either method. For the intraobserver variability evaluation, both methods were repeated once. Manual drawing of the LV epicardial and endocardial contours on the two sets of composite images from each method was done with an interval of at least 1 week in between. The first author with 6 years of experience in cardiovascular imaging set the window level and width of all images so that they were equal for all observers. The semi-automatic myocardial segmentation was then conducted on the two sets of previously generated composite images. The time spent for the generation of the composite images by the two methods, and for drawing the manual and semi-automatic myocardial contours, was recorded automatically by the software. RBP and JMvS had profound knowledge of short-axis myocardial anatomy and are further referred to as “non-radiologists”. Myocardial contour agreement within and between observers using manual drawing and semi-automatic segmentation were assessed by the dice similarity coefficient (DSC):

3 $${\text{DSC = }}(A,B){ = }\frac{ 2(A \cap B )}{(A + B)}$$

where *A* and *B* represent the contour regions and ∩ and + represent the intersection and addition between regions, respectively, with a minimum DSC value of 0 which indicates no contour agreement and a maximum value of 1 in case of total overlap [30].

### Statistical analysis

Agreements on myocardial area and contour similarity (DSC) were assessed and presented as median ± median absolute deviation (MAD). A paired Wilcoxon test was used to compare the DSC agreement of manual drawing and semi-automatic segmentation conducted on the composite images generated by the two methods. The Bland–Altman analysis [38] of segmental T2* reproducibility between and within observers as assessed by manual and semi-automatic segmentation is presented by mean difference ± limit of agreement (LoA). The LoA is defined as 1.96× standard deviation (SD) of the difference. The identification of a segment with the minimum T2* value per slice was based on the result of semi-automatic segmentation of the composite images generated by method 2 (that turned out to have highest performance in contour reproducibility). Based on this quantification, two groups with and without local iron deposition were identified (T2* value ≤20 and >20 ms, respectively) [39, 40].

Intraobserver and interobserver reproducibility of manual and semi-automatic segmentation using the two methods was assessed in the following groups; all observers, radiologist, non-radiologist, and in the pathology groups of with and without local iron deposition. Segmental T2* analysis between and within observers was performed in the same way as described for the myocardial contours. Variability was presented by the CoV, expressed as a percentage. Reliability was presented as the two-way random intra-class correlation coefficient (ICC), evaluating absolute agreement. The Friedman test was used to assess the reproducibility of optimal TE image selection and CNR improvement of the composite image between observers. IBM SPSS Statistics software version 20 (IBM Corporation, Somers, NY, USA) was used to perform all statistical analyses, with *P* < 0.05 being considered as statistically significant.

## Results

### CNR based on composite image generation

In this study, two different methods of selecting the myocardial surrounding regions used in the generation of the contrast-optimized composite images were evaluated. Manual input per patient (three short-axis slices) to generate the composite image by method 1 was small compared with method 2 (mean ± SD of 41.19 ± 4.80 s vs. 96.12 ± 13.98 s, *P* < 0.01). Thus, two or three images from the MGE series were generally selected and weighted in a ratio of two to one (two images) or equally (three images) [30] to produce the composite images (43.52 and 55.50% in method 1, and 50.49 and 49.51% in method 2, respectively). In method 1, the longest TE image (8th TE of 18.20 or 18.86 ms, scanned by the Avanto or Aera scanner, respectively) was commonly selected as a representation of maximum CNR between LV myocardium and both LVBP (65.69%), and RVBP (32.60%), while the image of the second TE (4.82 or 5 ms by the Avanto or Aera scanner, respectively) was chosen for maximum CNR between LV myocardium and lung (47.79%). The same pattern was observed in the image selection in method 2, where the maximum CNR between the LV myocardium and both LVBP and RVBP were observed at the longest TE (72.79 and 54.17%, respectively), and between the LV myocardium and lung at the shortest TE (2.59 or 2.69 ms by the Avanto or Aera scanner, respectively; 83.09%).

In method 2, reproducibility of selecting the same MGE images for maximum CNR between the LV myocardium and its surroundings was higher than in method 1, as evidenced by higher ICC when using method 2 compared to method 1 (0.981 vs. 0.897 between the LV myocardium and LVBP, 0.951 vs. 0.877 between the LV myocardium and RVBP, and 0.947 vs. 0.862 between the LV myocardium and lung). Only in method 1 was a significant difference was found between observers in MGE image selection for maximum CNR between the LV myocardium and RVBP (*P* < 0.05). Using method 2 for generation of the composite image, CNR gains (relative to the highest CNR of any original MGE image) were consistently higher as compared to method 1 between the LV myocardium and LVBP [mean 80.87%, 95% confidence interval (CI) of 74.89–86.85% vs. mean 45.32%, 95% CI of 39.23–51.42%], between the LV myocardium and RVBP (mean 94.71%, 95% CI of 86.79–102.63% vs. mean 78.36%, 95% CI of 69.69–87.04%), and between the LV myocardium and lung (mean 69.19%, 95% CI of 62.94–75.44% vs. mean 91.92%, 95% CI of 84.32–99.51%). On average, the composite images improved contrast between the LV myocardium and its surroundings by 81.48% (95% CI of 77.55–85.43%) using method 2 and by 71.87% (95% CI of 67.40–76.34%) using method 1 above the maximum CNR at any single TE.

### Reproducibility of myocardial contours

The observers produced the contours by manual drawing and semi-automatic segmentation (three short-axis slices per patient) in, on average, 73.82 ± 13.70 s and 14.26 ± 0.74 s, respectively (*P* < 0.001). Adding up the composite image generation time to that of the contour revealed that the semi-automatic segmentation method took less time than manual drawing by both method 1 (mean ± SD of 55.58 ± 4.90 vs. 107.13 ± 12.01 s, *P* < 0.001), and method 2 (mean ± SD of 110.25 ± 13.56 vs. 177.83 ± 27.93 s, *P* < 0.001).

Intraobserver reproducibility analysis of 51 short-axis slices yields a total of (51 × 4 = 204) DSCs contours while, for interobserver reproducibility, the total DSCs becomes (51 × 24 = 1224) DCSs. For all observers, intraobserver reproducibility of myocardial contours on contrast-optimized composite images generated by the two methods improved when using the semi-automatic segmentation compared to manual drawing (DSC of 0.86 ± 0.05 vs. 0.83 ± 0.04, *P* < 0.001 by method 1 and DSC of 0.86 ± 0.05 vs. 0.81 ± 0.03, *P* < 0.001 by method 2). The same trend of DSCs improvement by using the semi-automatic segmentation on method 1 (Table 1) and method 2 (Table 2) was also found within observers of different experience level and when the data were differentiated into short-axis slices with segmental T2* ≤ 20 and >20 ms. The only exception is that DSC improvement within non-radiologists lacked significance on method 1 (*P* > 0.05). Between all observers, interobserver reproducibility was improved by using the semi-automatic segmentation compared to manual drawing when using both method 1 (*P* < 0.001) and method 2 (*P* < 0.001) and likewise in all subgroups with better DSCs generally acquired when using the semi-automatic segmentation rather than manual drawing (Tables 1 and 2).

**Table 1:** Intraobserver and interobserver reproducibility of myocardial region determination and segmental T2* quantification assessed by using manual drawing and semi-automatic segmentation on contrast-optimized composite images by using method 1 at all slices on all patients

	nsa	Myocardial region determination						ns	Segmental T2* quantification			
		Area (median ± MAD%)^a^		*P*	DSC (median ± MAD)		*P*		Mean difference ± LoA (ms)		CoV (%)	
		M	SA		M	SA			M	SA	M	SA
Intraobserver reproducibility examined between												
Radiologist	102	83 ± 25	100 ± 21	<0.001	0.80 ± 0.05	0.86 ± 0.05	<0.001	544	−0.34 ± 6.27	−0.05 ± 5.95	14.02	12.67
Non-radiologist	102	113 ± 29	99 ± 24	<0.001	0.86 ± 0.03	0.86 ± 0.05	>0.05	544	−0.06 ± 6.09	−0.04 ± 6.46	13.25	13.59
	204	100 ± 26 (1340.91 ± 349.22 mm^2^ )	100 ± 23 (1272.16 ± 290.82 mm^2^ )	>0.05								
All observers on slices with minimum segment T2* ≤ 20 ms	152	104 ± 29	103 ± 21	<0.05	0.83 ± 0.04	0.86 ± 0.04	<0.01	832	−0.20 ± 5.96	−0.10 ± 5.91	14.24	13.53
All observers on slices with minimum segment T2* > 20 ms	52	85 ± 21	91 ± 25	>0.05	0.82 ± 0.05	0.83 ± 0.06	<0.05	256	−0.21 ± 6.86	0.12 ± 7.09	12.14	12.06
	204	100 ± 26 (1340.91 ± 349.22 mm^2^)	100 ± 23 (1272.16 ± 290.82 mm^2^)	>0.05								
Interobserver reproducibility examined between												
Radiologist	204	86 ± 25	100 ± 22	<0.001	0.75 ± 0.05	0.84 ± 0.05	<0.001	1088	−0.19 ± 8.61	0.39 ± 8.03	19.28	17.08
Non-radiologist	204	117 ± 30	100 ± 24	<0.001	0.81 ± 0.04	0.85 ± 0.05	<0.001	1088	1.18 ± 8.67	−0.08 ± 8.04	18.87	16.93
	408	100 ± 26 (1314.14 ± 337.05 mm^2^)	100 ± 23 (1297.11 ± 296.90 mm^2^)	<0.05								
All observers on slices with minimum segment T2* ≤ 20 ms	912	105 ± 29	104 ± 21	<0.001	0.77 ± 0.05	0.85 ± 0.05	<0.001	4992	−0.23 ± 8.53	−0.04 ± 7.33	20.37	16.77
All observers on slices with minimum segment T2* > 20 ms	312	92 ± 19	90 ± 24	0.056	0.76 ± 0.05	0.83 ± 0.06	<0.001	1536	−0.37 ± 8.04	−0.44 ± 8.19	14.22	13.92
	1224	100 ± 27 (1311.71 ± 353.48 mm^2^)	100 ± 23 (1292.85 ± 296.90 mm^*2*^)	<0.001								

*nsa* number of short-axis slices, *ns* number of segment, *DSC* dice similarity coefficient, *MAD* median absolute deviation, *LoA* limit of agreement, *CoV* coefficient of variation, *M* manual drawing, *SA* semi-automatic segmentation

^a^The percentage of myocardial areas were relative to the average area (measurements in mm^2^ were expressed inside bracket) measured per observer and pathology group

**Table 2:** Intraobserver and interobserver reproducibility of myocardial region determination and segmental T2* quantification assessed by using manual drawing and semi-automatic segmentation on contrast-optimized composite images by using method 2 at all slices on all patients

	nsa	Myocardial region determination						ns	Segmental T2* quantification			
		Area (median ± MAD%)^a^		*P*	DSC (median ± MAD)		*P*		Mean difference ± LoA (ms)		CoV (%)	
		M	SA		M	SA			M	SA	M	SA
Intraobserver reproducibility examined between												
Radiologist	102	86 ± 24	99 ± 27	0.001	0.80 ± 0.03	0.85 ± 0.04	<0.001	544	−0.004 ± 5.97	−0.09 ± 5.65	13.36	11.99
Non-radiologist	102	111 ± 29	101 ± 26	<0.001	0.83 ± 0.04	0.88 ± 0.06	<0.001	544	0.26 ± 6.50	0.15 ± 5.69	14.31	12.09
	204	100 ± 26 (1319.62 ± 348.00 mm^2^)	100 ± 27 1247.83 ± 330.97 mm^2^)	0.01								
All observers on slices with minimum segment T2* ≤ 20 ms	152	103 ± 32	104 ± 23	<0.05	0.81 ± 0.04	0.86 ± 0.05	<0.001	832	0.14 ± 6.30	0.03 ± 5.70	15.19	13.14
All observers on slices with minimum segment T2* > 20 ms	52	92 ± 19	96 ± 26	>0.05	0.83 ± 0.02	0.86 ± 0.05	0.001	256	0.08 ± 6.06	0.03 ± 5.58	10.70	9.46
	204	100 ± 26 1319.62 ± 348.00 mm^2^)	100 ± 27 (1247.83 ± 330.97 mm^2^)	0.01								
Interobserver reproducibility examined between												
Radiologist	204	87 ± 25	100 ± 27	<0.001	0.74 ± 0.04	0.83 ± 0.05	<0.001	1088	−0.40 ± 7.14	0.58 ± 7.15	15.99	15.17
Non-radiologist	204	112 ± 28	100 ± 26	<0.001	0.80 ± 0.04	0.86 ± 0.07	<0.001	1088	0.11 ± 7.51	0.03 ± 6.36	16.53	13.53
	408	100 ± 27 (1320.23 ± 350.44 mm^2^)	100 ± 26 (1218.63 ± 321.84 mm^2^)	<0.001								
All observers on slices with minimum segment T2* ≤ 20 ms	912	103 ± 29	103 ± 26	<0.001	0.77 ± 0.04	0.84 ± 0.05	<0.001	4992	−0.25 ± 7.16	0.14 ± 6.91	17.28	15.91
All observers on slices with minimum segment T2* > 20 ms	312	94 ± 19	98 ± 28	<0.05	0.76 ± 0.04	0.85 ± 0.05	<0.001	1536	−0.52 ± 7.33	0.10 ± 6.41	12.95	10.87
	1224	100 ± 27 (1313.54 ± 348.61 mm^2^)	100 ± 26 (1224.71 ± 323.06 mm^2^)	<0.001								

*nsa* number of short-axis slices, *ns* number of segment, *DSC* dice similarity coefficient, *MAD* median absolute deviation, *LoA* limit of agreement, *CoV* coefficient of variation, *M* manual drawing, *SA* semi-automatic segmentation

^a^The percentage of myocardial areas were relative to the average area (measurements in mm^2^ were expressed inside bracket) measured per observer and pathology group

**Fig. 6:**
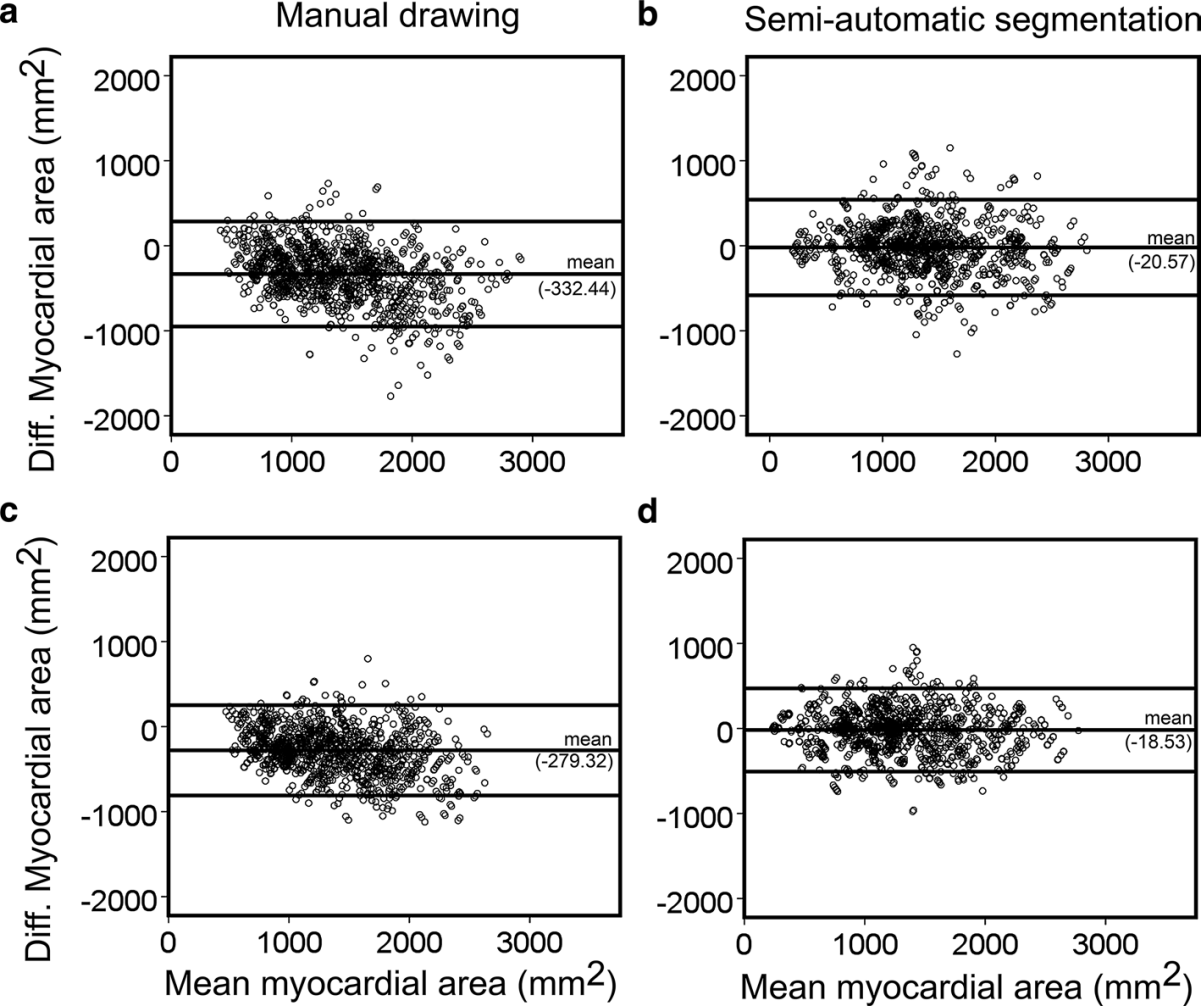
Bland–Altman plots between all observers assessing myocardial area on contrast-optimized composite images generated by method 1 on 17 patients at apical, mid-ventricular and basal locations (a total of 1224 short-axis slices) using manual drawing (**a)** and semi-automatic segmentation (**b)** and by method 2 using manual drawing (**c)** and semi-automatic segmentation (**d**)

In the first columns of Tables 1 and 2, LV myocardial areas measured by manual drawing and semi-automatic segmentation are presented in percentages of the average area per observer and pathology group, with the collective areas in each group were also expressed in mm^2^ between brackets. The radiologists tended to get smaller areas (83–87%) by the manual drawing with the two methods than the non-radiologists (111–117%). This variation is reduced by the semi-automatic segmentation with the two methods as performed by the radiologists (99–100%) and the non-radiologists (99–101%). Measurement bias of the LV myocardial area between all observers was also reduced by using the semi-automatic segmentation as compared to manual drawing (Fig. 6), both by using method 1 (mean difference ± LoA; CoV of −20.57 ± 562.43 mm^2^; 21.60% vs. −332.44 ± 617.02 mm^2^; 22.88%), and method 2 (mean difference ± LoA; CoV of −18.53 ± 489.22 mm^2^; 19.30% vs. −279.32 ± 530.24 mm^2^; 19.88%). A similar trend between all observers was found for slices with (mean difference ± LoA; CoV of −18.45 ± 521.04 mm^2^; 20.05% vs. −285.92 ± 553.45 mm^2^; 20.12%) and without the presence of local iron loading (mean difference ± LoA; CoV of −18.75 ± 382.00 mm^2^; 16.26% vs. −260.02 ± 454.37 mm^2^; 18.74%).

### Reproducibility of segmental myocardial T2*

Fifty-one short-axis slices of 17 patients at apical, mid-ventricular, and basal locations generated a dataset containing 272 myocardial segments according to the AHA 16-segment model. Evaluation of minimum segmental T2* values ≤20 ms revealed 38 slices with local iron deposition on 10 apical (eight at anterior, one at septal, seven at inferior, and five at lateral segments), 13 mid-ventricular (eight at anterior, two at anteroseptal, four at inferoseptal, six at inferior, eight at inferolateral, and four at anterolateral segments), and 15 basal locations (nine at anterior, five at anteroseptal, two at inferoseptal, four at inferior, eight at inferolateral, and five at anterolateral segments). Bland–Altman analysis of segmental T2* quantification using the manual drawing and semi-automatic segmentation is shown in Tables 1 and 2 for the assessment by methods 1 and 2.

In general, compared to method 1, segmental T2* quantification on the contrast-optimized composite image generated by method 2 shows lower LoA and CoV in intraobserver and interobserver groups assessed both by the semi-automatic segmentation and manual drawing. When intraobserver and interobserver variation is considered for all observers, lower LoA and CoV of T2* are obtained by the semi-automatic segmentation than by manual drawing generated both by method 1 and method 2. Consistent reductions of LoA and CoV of T2* were also found in intraobserver and interobserver subgroups by using the semi-automatic segmentation compared to manual drawing on method 2, while in method 1, inconsistent reductions were found in intraobserver variance between non-radiologists. Focusing on intraobserver and interobserver variation of T2* quantification in the global LV heart as compared with the mid-ventricular septum (Table 3) evaluation shows that the semi-automatic segmentation produces lower observer variability of T2* quantification compared to manual drawing in both global LV and mid-ventricular septal with the least spread when using method 2.

**Table 3:** Global and mid-ventricular myocardial T2* coefficient of variance (CoV) of all patients assessed within and between all observers using manual drawing and semi-automatic segmentation on contrast-optimized composite images generated by two methods

Segmental analysis	ns	Intraobserver		ns	Interobserver	
		Manual	Semi-automatic		Manual	Semi-automatic
		T2* CoV (%)	T2* CoV (%)		T2* CoV (%)	T2* CoV (%)
Method 1						
Global myocardium	1088	13.64	13.14	6528	18.57	15.97
Mid-ventricular septal	136	9.73	7.83	816	14.92	11.20
Method 2						
Global myocardium	1088	13.86	12.05	6528	16.01	14.43
Mid-ventricular septal	136	10.87	9.73	816	11.89	9.68

*ns* number of segment, *CoV* coefficient of variation

**Fig. 7:**
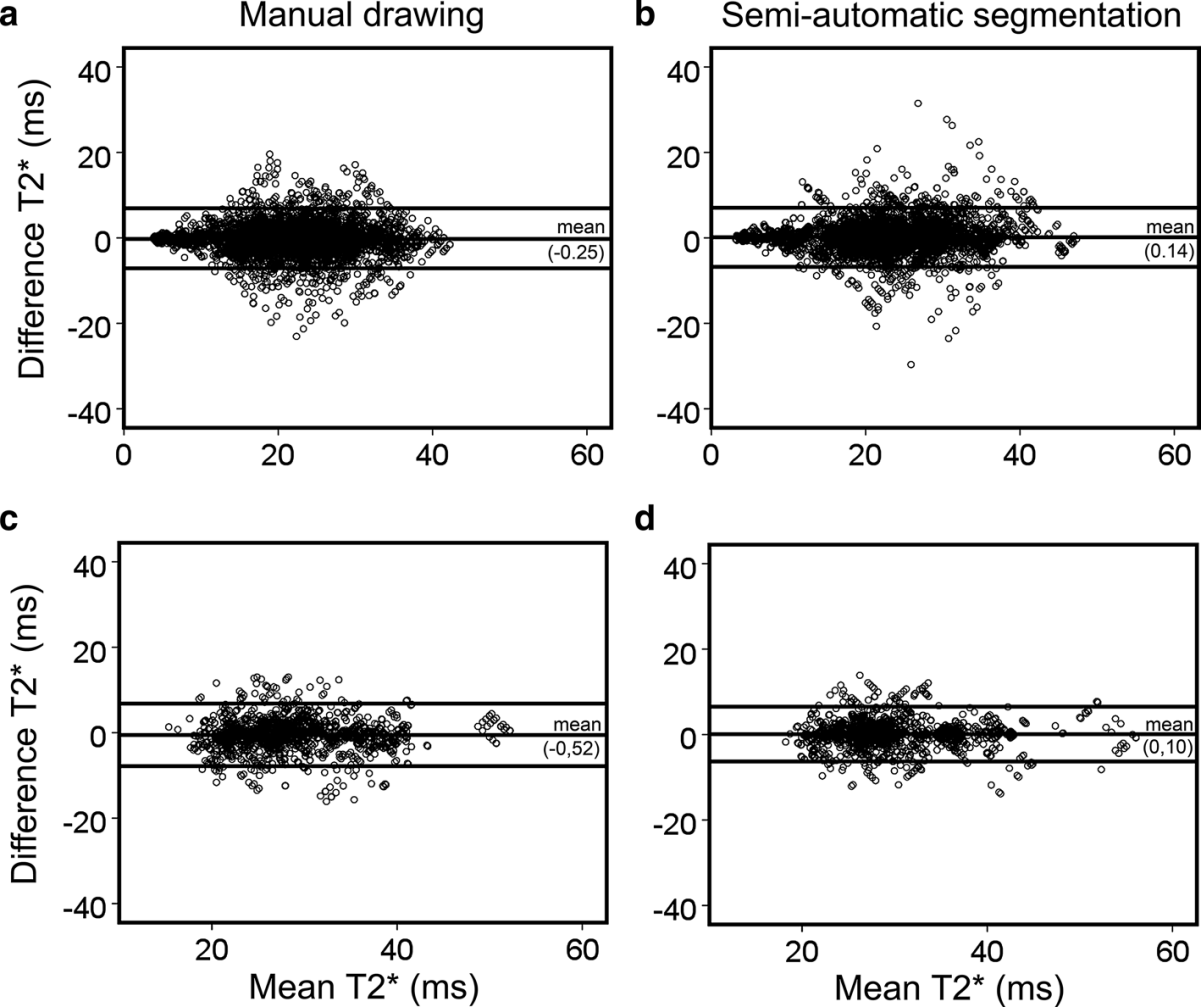
Bland–Altman plots between all observers assessing myocardial T2* on short-axis slices by manual drawing (**a)** and semi-automatic segmentation (**b)** on contrast-optimized composite image generated by method 2 in the presence of at least a myocardial segment with minimum T2* ≤ 20 ms (4992 segments) and by manual drawing (**c)** and semi-automatic segmentation (**d)** in the absence of any segment with minimum T2* ≤ 20 ms (1536 segments)

In the analysis of myocardial T2* ≤ 20 ms as an indication of iron deposition, consistent reductions of LoA and CoV of segmental T2* within and between all observers were found on the semi-automatic segmentation compared to manual drawing assessed on both methods with the same trends to those observed for segmental T2* > 20 ms. The Bland–Altman plots between all observers quantifying segmental T2* ≤ 20 and >20 ms by using the semi-automatic segmentation and manual drawing on method 2 are shown in Fig. 7.

## Discussion

This study shows that semi-automatic myocardial segmentation improves reproducibility within and between observers in defining myocardial area as compared to manual drawing with an almost five times faster post-processing time without significant elimination of pixels at the myocardial borders. This result is important to achieve reproducible and reliable myocardial T2* quantification, especially for thalassemia and suspected cardiomyopathy patients where evidence shows that early stage of iron deposition starts from the epicard [11, 13, 14, 41, 42]. Contour reproducibility is beneficial in the clinical evaluation of patients with iron loading indication, where our novel procedure reduces the variability in segmental myocardial T2* quantification (Table 2). The T2* approach presented in this study is not limited to mid-ventricular septum analysis, but also successfully applied for global analysis identifying local heterogeneity of iron deposition on different locations of the LV heart. This finding indicates that the semi-automatic segmentation can replace the standard manual approach (Table 3). Even though MGE acquisition was done on two different scanners in this study, other studies have confirmed the reproducibility of myocardial T2* data from different centers operating different scanners [43, 44]. The LV myocardial contour improvement in this study is achieved by using the contrast-optimized composite images generated by method 2, which, on average, improves the CNR between myocardium and its surrounding regions by 81.48%.

The use of contrast-optimized composite images in improving the CNR between myocardium and its surroundings has been shown previously [30]. In that study, manual location and drawing of ROIs in the surroundings of the LV myocardium served to select those three images from the MGE series to be merged into composite images based on maximum CNRs. It was the intention in this study to replace that rather subjective approach with a more systematic approach so that a more reproducible TE image combination can be produced.

In this study, two methods for generating the combination of three TE images were, therefore, assessed and evaluated. Although method 1 was even faster (*P* < 0.001), method 2, with a more objective approach, produced higher reliability (higher ICC) with a higher percentage rate of the same image selection. This improved the CNR gain between the LV myocardium and its surroundings on the composite images compared to method 1 (81.48 vs. 71.87%). The drawback of using method 1 in producing a reliable MGE image selection is in the use of an anterior RV–LV insertion point as reference to create the six segments and the fixed positions of RVBP and lung ROIs at the edge of the segments, rendering the procedure more observer-dependent and eliminating the flexibility to adapt the lung (Fig. 2c) and RV (Fig. 2b) sizes. This drawback influences the generated composite image due to remaining variation in the selection of images providing optimum contrast between the LV myocardium and RVBP and between the LV myocardium and lung. Our results show that the generation of a composite image is made by mostly combining images at the first and the last TE, similar to previous study [30]. However, higher repeatability was achieved in this study when comparing the percentages of TE image selection (proven also by high ICCs) implying superiority of our approach (method 2) in generating contrast-optimized composite images compared with the previous study.

Unlike the black blood MGE series [26], blood signal artifacts and partial volume effects in bright blood MGE series create signal inhomogeneity in LVBP, making the segmentation of LVBP contours challenging. In this study, the subtraction of the shortest TE image from that with optimum TE between the LV myocardium and LVBP was proven as an effective technique to reduce inhomogeneity by creating a low signal intensity image. Due to the presence of artefacts in the bright blood mode, the homogeneous signal at the center of the LVBP is not circularly shaped and, therefore, the circular Hough transformation proposed by Zheng et al. [26] cannot be implemented as a start for LVBP area detection. Therefore, we combined *k*-means clustering together with VFC active contours to encounter the LVBP border detection problem on all short-axis images acquired not only on mid-ventricular but also on apical and basal locations. Others reported the use of watershed segmentation after imaging morphological processes on the bright blood mode to acquire a mask image for the LVBP boundary detection by Geodesic active contours and Level set [27]. But the combination of these methods still introduces a high false detection of LVBP centroid leading to false endocardial contour determination. As proposed by Lynch et al. [32], the first step in contouring is the key to success for a robust active contour segmentation. The use of a manually drawn LV myocardial wall contour as the initial active contour in this study did influence the robustness of LVBP determination generated by different observers, especially in apical locations, as seen in Fig. 5b, d. Nevertheless, variability in contours was lower than that produced with manual contour determination (Fig. 5).

We introduced a new technique to determine the epicardial border of LV myocardium by using mean signal intensity information of the LV myocardial wall located on several radial pixel’s segments and layers. CoV assessment and *k*-means clustering were then applied in the combination of these segments and layers to determine the epicardial border. The CoV was proven to be effective in determining the lateral border, while the *k*-means clustering was able to determine the anterior, septal, and posterior borders. By further automated correction procedures, low-contrast problems interfering with the depiction of the epicardial border at septal, anterior, and posterior locations could be solved. Prior knowledge of the LV myocardial wall was used in this technique to ensure the effectiveness of the clustering method in determining the epicardial border location.

The semi-automatic myocardial segmentation in this study reduces the post-processing time to several orders of magnitude shorter than that reported elsewhere [27] and yields higher intraobserver and interobserver reproducibility as compared to manual drawing, leading to lower variability in myocardial T2* quantification regardless of the presence or absence of iron loading pathology (T2* ≤ 20 and >20 ms). The results show that the semi-automatic segmentation method produces roughly the same area of myocardium compared to manual drawing (Tables 1 and 2, area of LV myocardial region determination) with the advantage of lower variability in area measurement (lower SD in general) and substantial reduction of measurement bias between observers (Fig. 6). Compared to other study [30], here higher observer variability in T2* quantification is influenced by subjective input, reflecting the observer’s background in defining the myocardial area and is reduced by using the semi-automatic segmentation (higher DSC with *P* < 0.001). Another reason is the heterogeneity of myocardial T2* in patients with borderline iron deposition in this study.

Consistence of areas regardless of the observer’s background make the segmentation method in this study a reliable LV myocardial area detection method for T2* quantification in the presence and absence of iron deposition (Figs. 5, 6). Our results are consistent with another study showing that radiologists tend to create tighter myocardial area definition than automatic detection [27]. Combining LV myocardial area analysis with contour shape analysis (DSC) in this study revealed that the semi-automatic segmentation produces more reproducible LV myocardial contours as compared to manual drawing while maintaining myocardial pixels near the endocardial and epicardial borders. This indicates that the possible loss of biologic information contained in low-T2* pixels at the periphery is negligible with the semi-automated segmentation, especially method 2. In the end, the global and mid-ventricular septal myocardial T2* quantification (Table 3) and the semi-automatic segmentation lowered variability between observers.

This study has a limitation in that the validation of the semi-automatic segmentation was done on a small sample of patient data in accordance with previous methodological studies [19, 27]. The next step will be to validate the new method in a larger patient group in clinical practice. The monoexponential fitting model with constant offset used in this study yields only a small systematic bias for patients with borderline iron loading [4] compared to other fitting methods recently used [9, 16, 45]. The use of a combined truncation method with image corrections [9, 16, 45] might be considered in future work. Here, local susceptibility artifacts might have influenced T2* heterogeneity even though its presence, after correction, is known to not significantly affect global heart T2* heterogeneity [18, 46]. Another suggestion made in the literature is the use of susceptibility correction [15–18]. Maximum myocardial layer expansion in the semi-automatic segmentation was set at 17 mm from the epicardial border, an optimization which in the application for hypertrophic cardiomyopathy patients can only be applied for mild LV wall thickening identification [47]. Prior pathology information of images with more severe hypertrophic condition could be added in the segmentation procedure to customize the myocardial layer expansion on specific myocardial thickening regions, i.e. >30 mm [48]. Even though method 2 has shown an improvement in selecting MGE images for the composite combination, the required manual drawing of RVBP and lung areas still is time-consuming. Manual definition of the myocardial wall to start the semi-automatic segmentation process causes some variation in the results within and between observers and increases the time expenditure for contour generation. Here, the developed semi-automatic segmentation method was only applied on a bright blood MGE image series showing poor contrast differences between the myocardium and its surroundings. Successful implementation of an entirely automatic approach to select the region by using boundary-based or region-based methods [19, 20] to shorten the process, and further improve the reproducibility of bright blood MRI might be a next step. In the meantime, it will be of interest to further validate our segmentation method in a black blood MGE series in comparison with alternatives provided by others.

## Conclusion

In conclusion, the proposed semi-automatic myocardial segmentation as assessed on contrast-optimized composite images provides comparable and reproducible T2* quantification faster than manual drawing, and can be applied in clinical practice for global heart and mid-ventricular septum analysis. The effectiveness of the segmentation is influenced by the contrast difference between the myocardium and its surrounding tissues where, in this study, a CNR gain of, on average, 81.48% was achieved. In iron loading assessment, the proposed segmentation method leads to more consistent and less user-dependent myocardial detection areas resulting in better reproducibility of T2* quantification.
